# Effects of resection volume on postoperative micturition symptoms and retreatment after transurethral resection of the prostate

**DOI:** 10.1007/s00345-023-04628-0

**Published:** 2023-10-03

**Authors:** Seung Han Shin, Kwang Suk Lee, Kyo Chul Koo, Kang Su Cho, Chang Hee Hong, Byung Ha Chung, Hyun Soo Ryoo, Jae Hyun Ryu, Yun Beom Kim, Seung Ok Yang, Jeong Kee Lee, Tae Young Jung, Jeong Woo Yoo

**Affiliations:** 1 Department of Urology, Veterans Health Service Medical Center, 53 Jinhwangdo-Ro 61-Gil, Gangdong-Gu, Seoul, 05368 Republic of Korea; 2 Department of Urology, Gangnam Severance Hospital, Yonsei University College of Medicine, Seoul, Republic of Korea

**Keywords:** Benign prostatic hyperplasia, International prostatic symptom score, Resection volume, Transurethral resection of the prostate

## Abstract

**Purpose:**

Despite advances in technology, such as advent of laser enucleation and minimally invasive surgical therapies, transurethral resection of the prostate (TURP) remains the most widely performed surgical technique for benign prostatic hyperplasia (BPH). We evaluated resection volume (RV)-derived parameters and analyzed the effect of RV on post-TURP outcomes.

**Methods:**

This observational study used data from patients who underwent TURP at two institutions between January 2011 and December 2021 Data from patients with previous BPH surgical treatment, incomplete data, and underlying disease affecting voiding function were excluded. The collected data included age, prostate-specific antigen, transrectal ultrasound (TRUS)- and uroflowmetry-derived parameters, RV, perioperative laboratory values, perioperative International Prostatic Symptom Score (IPSS), follow-up period, retreatment requirements and interval between the first TURP and retreatment.

**Results:**

In 268 patients without prior BPH medication, there were no differences in prostate volume (PV), transitional zone volume (TZV), or RV according to IPSS. A total of 60 patients started retreatment, including medical or surgical treatment, within the follow-up period. There was a significant difference in RV/PV between the groups without and with retreatment respectively (0.56 and 0.37; *p* = 0.008). However, preoperative TRUS- and uroflowmetry-derived parameters did not differ between the two groups. Multiple linear regression analysis showed that RV (*p = 0.003*) and RV/TZV (*p* = 0.006) were significantly associated with differences in perioperative IPSS. In the multivariate logistic regression analysis, only RV/PV was correlated with retreatment (*p* = 0.010).

**Conclusion:**

Maximal TURP leads to improved postoperative outcomes and reduced retreatment rate, it may gradually become a requirement rather than an option.

## Introduction

Benign prostatic hyperplasia (BPH) is histologically evident mainly in the transition zone and results from the proliferation of epithelial cells and smooth muscle [1]. It has a prevalence of 10% in 40 s-year-old men and 50% in 50 s-year-old men [2], which is gradually increasing in Asia owing to the influence of westernized eating habits, an increase in average life expectancy, and an aging society. This severely impacts the quality of life (QoL) of elderly men. Clinically, physicians are encountering more large prostates in current times than in the past.

There are various treatments for severe benign prostatic hyperplasia, including medical or surgical managements and their combinations. Medical treatment is based on combination therapy, as demonstrated in the CombAT trials [3, 4]. In some selected patients, minimally invasive surgical therapies (MISTs), such as prostatic urethral lift (PUL) [5], water vapor thermal therapy, and prostatic artery embolization (PAE) [6], are possible. Additionally, there are several options for surgical treatments, such as photoselective vaporization of the prostate [7], holmium laser enucleation of the prostate (HoLEP) [8], and transurethral resection of the prostate (TURP) [1]. In HoLEP, which has recently been highlighted among surgical treatments, the entire adenoma is removed along the surgical capsule. However, in the case of TURP, maximal TURP (resection until the surgical capsule is exposed) and minimal TURP (resection for only tunneling) are performed at the surgeon’s discretion [9]. The difference between the outcomes of maximal and minimal TURP is also controversial [9, 10].

Approximately 30 years ago, when mainly monopolar TURP was performed, there were restrictions on the operation time due to post-TURP syndrome. At this time, several studies were conducted on the correlation between resection volume (RV)-derived parameters and TURP outcomes. After the introduction of bipolar TURP, there were less restrictions on the operation time; however, very few studies recently investigated the relationship between RV-derived parameters and the outcomes. Therefore, we evaluated RV-derived parameters according to the size of the prostate and analyzed the effect of RV on postoperative outcomes of bipolar TURP.

## Material and methods

### Ethics statement

This study was approved by the Institutional Ethics Committee (2021-0106-001), and all procedures were conducted in accordance with the ethical standards of the 1964 Declaration of Helsinki and its later amendments. The requirement for informed consent was waived because the study was based on retrospective and anonymous patient data and did not involve patient intervention or human tissue samples.

### Data collection

**Fig. 1:**
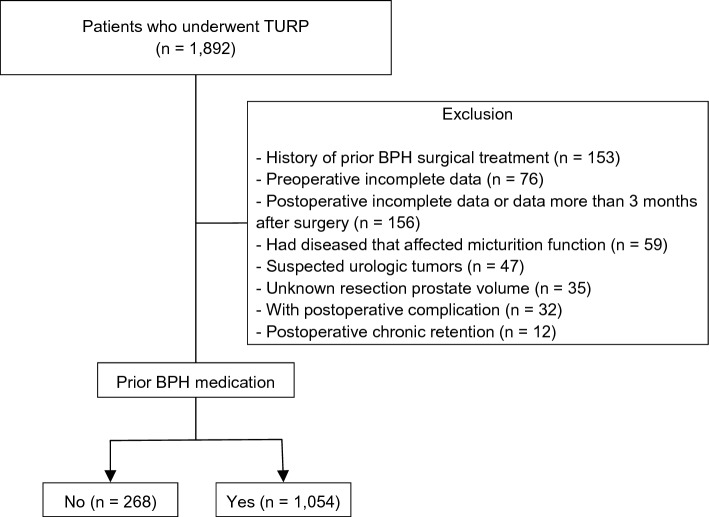
Study cohort flow diagram. BPH, benign prostatic hyperplasia; TURP, transurethral resection of the prostate

This observational study was based on data from patients who underwent TURP at two institutions between January 2011 and December 2021. Data were collected from their electronic medical charts. The number of excluded patients was calculated and recorded in the order of each exclusion criterion and there were many patients with overlapping exclusion criteria. The exclusion criteria were as follows (Fig. 1): (1) patients with a history of prior BPH surgical treatment (*n = *153); (2) incomplete data regarding preoperative international prostatic symptom score (IPSS), transrectal ultrasound (TRUS)-derived parameters, uroflowmetry derived parameters (*n = *76), and incomplete postoperative IPSS data or the data had elapsed 3 months after surgery (*n = *156); (3) patients had diseases that affected micturition function such as neurologic disease or uncontrolled diabetes mellitus (*n = *59); (4) patients with suspected urologic tumors (bladder, prostate, and kidney tumors) (*n = *47); (5) unknown resection prostate volume (*n = *35); (6) patients with postoperative complications such as infection, incontinence, and clot retention (*n = *32); (7) patients had perioperative chronic retention (post-void residual volume > 300 mL) (*n = *12). Of the total 1892 patients, 570 were excluded. Among the remaining 1322 patients, 268 without prior BPH medication history and 1,054 with a BPH medication history were divided into subgroups and analyzed. Data regarding the following variables were collected: age, prostate-specific antigen, TRUS-derived parameters, uroflowmetry-derived parameters (peak flow rate [Qmax], voided volume, and post-void residual volume), RV, perioperative laboratory values (hemoglobin, platelet, sodium, and potassium), perioperative IPSS, follow-up period, requirement of retreatment (including medical or surgical treatment), and interval between the first TURP and retreatment.

### TRUS derived parameters and definitions

The status of the patients’ micturition symptoms was stratified using the IPSS questionnaire (mild, 1–7 points; moderate, 8–19 points; and severe, 20–35 points). The measured parameters were prostate volume (PV), transition zone volume (TZV), intravesical prostatic protrusion (IPP), prostatic urethral angle (PUA), and prostatic urethral length. The PV and TZV were calculated using the following prostate ellipsoid formula: (height × width × length × *π*/6) [11, 12]. The transitional zone index was calculated as TZV/PV. The IPP was defined as the vertical distance from the tip of the intravesical prostatic protrusion to the base of the bladder neck in the parasagittal plane of TRUS [12]. PUA was defined as the larger angle consisting of the two planes of the proximal and distal prostatic urethra on the parasagittal plane of TRUS, which was performed with minimal pressure from the transrectal probe to prevent PUA deformity [13]. Prostatic urethral length was defined as the sum of the proximal prostatic urethra, including the IPP, and the distal prostatic urethra [14]. The RV data were collected based on the specimen pathology report by the department of pathology at each institution. The follow-up period was calculated from the date of the first TURP to the date of the last outpatient clinic visit.

### TURP procedure

In general, patients undergo urinalysis and urine culture before surgery. If bacteria were detected in the urine culture, appropriate antibiotics are administered to confirm a negative culture result before proceeding with the surgery. The choice of antibiotics and duration of administration was at the discretion of the urologist. In cases where bacteria were not identified in the urine culture, a first-generation cephalosporin was used as a prophylactic antibiotic for 3 days, starting from the day of surgery.

TURP was performed on a lithotomy position under general anesthesia or spinal anesthesia, depending on the urologist’s preference. Several bipolar resectoscopes (Olympus, Tokyo, Japan; Richard Wolf GmbH, Knittlingen, Germany; Karl Storz, Tuttlingen, Germany) were used for the procedures. The choice of electrodes, such as loop-, mushroom-, and roller-electrodes depended on the operator. All TURPs were performed by experienced operators with a history of more than 100 cases of TURP. After surgery, the resected tissues were squeezed and sent to the department of pathology. Foley catheters were indwelling for 2–7 days after surgery. Once the urine color becomes clear, the catheter is removed, and the patient can be discharged.

### Study endpoints

The main study endpoint is to identify perioperative predictors that affect patient subjective symptom improvement and retreatment after TURP. The secondary endpoint is to determine the optimal cut-off value for significant predictors identified in the main study endpoint.

### Statistical Analyses

All values are expressed as numbers (%) or mean ± standard deviation, as appropriate. Continuous variables are expressed as medians (interquartile range). The parameters were compared between patient groups using the Student’s *t* test for continuous variables and the chi-square test (Fisher’s exact test) for two or more variables. Univariate and multivariate linear regression analyses were performed to identify the independent predictors of IPSS severity and retreatment. All reported *p-values* are two-sided. A *p* value < 0.05 was considered statistically significant. All statistical tests were conducted using the SPSS software (version 25.0; SPSS, Chicago, IL, USA).

## Results

### Baseline patient characteristics

The baseline characteristics of whole patients according to prior BPH medication are presented in Table 1. There was no difference in the PV (*p* = 0.445), TZV (*p* = 0.532), or RV (*p* = 0.289) according to prior BPH medication (Table 1). The differences of perioperative platelet (*p* = 0.051), total IPSS (*p* = 0.098) and QoL (*p* = 0.065) are presented marginally significant (Table 1).

**Table 1:** Baseline characteristics of patients according to prior BPH medication

	Total	Without prior medication	With prior medication	*p*
No. of patients	1322	268 (20.27)	1054 (79.72)	
Age (years)	69.69 ± 8.01	70.81 ± 6.31	69.41 ± 8.95	0.954
PSA (ng/ml)	3.39 ± 2.68	3.58 ± 2.39	3.34 ± 2.71	0.781
IPSS–total	17.85 ± 7.89	19.00 ± 8.34	17.56 ± 7.29	0.159
IPSS–QoL	3.88 ± 1.87	3.91 ± 1.28	3.87 ± 2.02	0.334
TRUS derived parameters				
PV (cm^3^)	60.97 (40.98–82.11)	59.95 (40.93–79.60)	61.23 (41.34–82.34)	0.445
TZV (cm^3^)	36.93 (23.35–52.50)	35.10 (22.15–49.80)	37.39 (24.27–53.23)	0.532
TZI	0.60 (0.50–0.67)	0.58 (0.50–0.65)	0.61 (0.52–0.67)	0.511
IPP (mm)	3.85 ± 1.89	3.99 ± 1.86	3.82 ± 1.90	0.101
PUA (°)	137.68 ± 14.58	138.93 ± 13.62	137.36 ± 14.88	0.363
PUL (mm)	50.71 ± 10.92	49.63 ± 9.31	50.99 ± 11.27	0.682
UFR derived parameters				
*Q*max (mL/sec)	10.30 ± 6.20	10.64 ± 6.61	10.21 ± 5.95	0.715
V oided volume (mL)	164.39 ± 122.65	172.87 ± 111.58	162.23 ± 127.21	0.218
PVR (mL)	101.55 ± 102.20	96.52 ± 99.30	102.83 ± 102.32	0.198
Resection volume (cm^3^)	19.07 (11.60–25.63)	18.00 (11.00–26.00)	19.34 (11.68–24.83)	0.289
Resection volume/PV	0.32 (0.25–0.42)	0.31 (0.22–0.42)	0.32 (0.27–0.42)	0.981
Resection volume/TZV	0.52 (0.43–0.70)	0.55 (0.41–0.72)	0.51 (0.43–0.70)	0.366
Difference^a^				
Hb (g/dL)	0.56 ± 0.82	0.58 ± 0.90	0.56 ± 0.79	0.899
Platelet (10^3^/μ/L)	20.87 ± 28.56	13.10 ± 33.62	22.84 ± 28.14	0.051
Sodium (mmol/L)	− 0.44 ± 2.10	− 0.47 ± 2.36	− 0.43 ± 1.98	0.391
Potassium (mmol/L)	0.03 ± 0.53	0.04 ± 0.42	0.03 ± 0.57	0.779
IPSS–total	7.08 ± 6.19	6.19 ± 8.76	7.31 ± 5.24	0.098
IPSS–QoL	1.29 ± 1.60	1.05 ± 1.76	1.35 ± 1.55	0.065

Data are presented as number (%), mean ± standard deviation, and median (interquartile range)

*BPH* benign prostatic hyperplasia; *Hb* hemoglobin; *IPP* intravesical prostatic protrusion; *IPSS* International Prostate Symptom Score; *PSA* prostatic-specific antigen; *PUA* prostatic urethral angle; *PUL* prostatic urethral length; *PV* prostate volume; *PVR* post-void residual volume; *QoL* Quality of life; *Qmax* peak flow rate; *TZI* transition zone index; *TZV* transition zone volume

^a^The values obtained by subtracting the post-operative values from the pre-operative values

### Baseline patient characteristics in the group without prior BPH medication

The baseline patient characteristics in the group without prior BPH medication according to the severity of the total IPSS are presented in Table 2. There was no difference in the PV (*p* = 0.545), TZV (*p* = 0.779), or RV (*p* = 0.709) according to IPSS severity (Table 2). Table 3 presents the demographics of patients with or without retreatment, including medication or surgery. A total of 60 patients started retreatment, including medical or surgical treatment, within the follow-up period. The median duration from the first surgery to retreatment was 121.0 (37.25–349.00) days. In the group without retreatment, the median follow-up period was 1103.5 (673.25–1327.50) days. There was a difference in RV/PV between group without and with retreatment (0.56, 95% confidence interval [CI] 0.22–0.68 and 0.37, 95% CI 0.27 – 0.47, respectively; *p* = 0.008). However, the preoperative TRUS- and UFR-derived parameters did not differ between the two groups (Table 3).

**Table 2:** Baseline characteristics of the patients according to the severity of IPSS in the group without prior BPH medication

	Total	Mild	Moderate	Severe	*p*
No. of patients	268	42 (15.67)	106 (39.55)	120 (44.78)	
Age (years)	70.81 ± 6.31	72.73 ± 6.81	72.04 ± 4.59	69.22 ± 7.30	0.045
PSA (ng/ml)	3.58 ± 2.39	3.81 ± 1.44	3.52 ± 2.42	3.57 ± 2.59	0.941
IPSS–total	19.00 ± 8.34	5.00 ± 1.34	14.24 ± 3.13	26.59 ± 4.40	< 0.001
IPSS–QoL	3.91 ± 1.28	1.73 ± 1.27	3.57 ± 0.91	4.71 ± 0.83	< 0.001
TRUS derived parameters					
PV (cm^3^)	59.95 (40.93–79.60)	77.15 (44.65–85.40)	56.90 (40.05–71.85)	57.90 (42.50–75.70)	0.545
TZV (cm^3^)	35.10 (22.15–49.80)	43.70 (21.95–52.85)	32.65 (22.90–43.03)	35.10 (21.70–47.20)	0.779
TZI	0.58 (0.50–0.65)	0.55 (0.19–0.63)	0.60 (0.51–0.65)	0.57 (0.49–0.66)	0.731
IPP (mm)	3.99 ± 1.86	5.23 ± 3.19	4.15 ± 1.77	3.66 ± 1.69	0.169
PUA (°)	138.93 ± 13.62	141.02 ± 12.24	136.98 ± 13.67	140.43 ± 14.11	0.449
PUL (mm)	49.63 ± 9.31	55.38 ± 15.08	49.41 ± 9.10	48.78 ± 8.02	0.181
UFR derived parameters					
*Q*max (mL/sec)	10.64 ± 6.61	9.02 ± 4.18	10.70 ± 5.78	11.01 ± 7.76	0.677
Voided volume (mL)	172.87 ± 111.58	170.72 ± 76.57	175.21 ± 103.58	171.39 ± 127.16	0.987
PVR (mL)	96.52 ± 99.30	103.82 ± 124.54	88.49 ± 84.82	101.69 ± 105.86	0.816
Resection volume (cm^3^)	18.00 (11.00–26.00)	20.00 (7.00–24.00)	16.00 (10.25–24.08)	22.00 (11.75–29.00)	0.709
Resection volume/PV	0.31 (0.22–0.42)	0.24 (0.13–0.27)	0.33 (0.25–0.42)	0.38 (0.24–0.46)	0.107
Resection volume/TZV	0.55 (0.41–0.72)	0.46 (0.39–0.54)	0.49 (0.43–0.67)	0.59 (0.35–0.75)	0.127
Difference^a^					
Hb (g/dL)	0.58 ± 0.90	0.79 ± 0.85	0.50 ± 1.01	0.60 ± 0.82	0.614
Platelet (10^3^/μ/L)	13.10 ± 33.62	27.55 ± 21.33	9.71 ± 37.96	13.24 ± 30.89	0.285
Sodium (mmol/L)	− 0.47 ± 2.36	− 1.27 ± 2.87	− 0.14 ± 2.35	− 0.61 ± 2.26	0.306
Potassium (mmol/L)	0.04 ± 0.42	0.14 ± 0.35	-0.18 ± 0.43	0.07 ± 0.42	0.409
IPSS–total	6.19 ± 8.76	− 1.36 ± 4.63	1.63 ± 6.42	12.32 ± 7.36	< 0.001
IPSS–QoL	1.05 ± 1.76	− 0.18 ± 1.54	0.57 ± 1.58	1.80 ± 1.67	< 0.001

Data are presented as number (%), mean ± standard deviation, and median (interquartile range)

*BPH* benign prostatic hyperplasia; *Hb* hemoglobin; *IPP* intravesical prostatic protrusion; *IPSS* International Prostate Symptom Score; *PSA* prostatic-specific antigen; *PUA* prostatic urethral angle; *PUL* prostatic urethral length; *PV* prostate volume; *PVR* post-void residual volume; *QoL* Quality of life; *Qmax* peak flow rate; *TZI* transition zone index; *TZV* transition zone volume

^a^The values obtained by subtracting the post-operative values from the pre-operative values

**Table 3:** Baseline characteristics of the patients with or without retreatment in the group without prior BPH medication

	Total	Without retreatment	With retreatment	*p*
No. of patients	268	208 (77.61)	60 (22.39)	
Age (years)	70.81 ± 6.31	71.02 ± 6.85	70.90 ± 5.67	0.931
PSA (ng/ml)	3.58 ± 2.39	3.48 ± 2.30	3.92 ± 2.73	0.437
IPSS–total	19.00 ± 8.34	19.18 ± 8.75	18.42 ± 6.96	0.689
IPSS–QoL	3.91 ± 1.28	3.92 ± 1.32	3.88 ± 1.18	0.909
TRUS derived parameters				
PV (cm^3^)	59.95 (40.93–79.60)	60.35 (43.85–80.05)	56.55 (38.33–70.90)	0.389
TZV (cm^3^)	35.10 (22.15–49.80)	35.95 (21.53–51.45)	31.50 (22.65–44.60)	0.763
TZI	0.58 (0.50–0.65)	0.57 (0.50–0.65)	0.59 (0.51–0.67)	0.800
IPP (mm)	3.99 ± 1.86	4.04 ± 1.72	4.04 ± 2.14	0.998
PUA (°)	138.93 ± 13.62	138.39 ± 14.23	140.70 ± 11.33	0.442
PUL (mm)	49.63 ± 9.31	50.33 ± 9.93	50.44 ± 9.61	0.961
UFR derived parameters				
*Q*max (mL/sec)	10.64 ± 6.61	10.89 ± 7.29	9.75 ± 4.85	0.444
Voided volume (mL)	172.87 ± 111.58	172.87 ± 109.41	150.21 ± 106.12	0.349
PVR (mL)	96.52 ± 99.30	87.65 ± 112.93	131.50 ± 112.85	0.083
Resection volume (cm^3^)	18.00 (11.00–26.00)	21.50 (10.00–24.58)	18.00 (13.50–30.00)	0.200
Resection volume/PV	0.31 (0.22–0.42)	0.56 (0.22–0.68)	0.37 (0.27–0.47)	0.008
Resection volume/TZV	0.55 (0.41–0.72)	0.68 (0.37–0.75)	0.55 (0.41–0.73)	0.971
Difference^a^				
Hb (g/dL)	0.58 ± 0.90	0.44 ± 0.90	0.72 ± 1.09	0.157
Platelet (10^3^/μ/L)	13.10 ± 33.62	13.70 ± 36.51	13.43 ± 46.65	0.974
Sodium (mmol/L)	− 0.47 ± 2.36	− 0.64 ± 2.42	− 0.57 ± 2.24	0.875
Potassium (mmol/L)	0.04 ± 0.42	0.06 ± 0.43	0.03 ± 0.47	0.696
IPSS–total	6.19 ± 8.76	6.18 ± 9.14	6.23 ± 7.60	0.979
IPSS–QoL	1.05 ± 1.76	1.10 ± 1.84	0.92 ± 1.47	0.664

Data are presented as number (%), mean ± standard deviation, and median (interquartile range)

*BPH* benign prostatic hyperplasia; *Hb* hemoglobin; *IPP* intravesical prostatic protrusion; *IPSS* International Prostate Symptom Score; *PSA* prostatic-specific antigen; *PUA* prostatic urethral angle; *PUL* prostatic urethral length; *PV* prostate volume; *PVR* post-void residual volume; *QoL* Quality of life; *Qmax* peak flow rate; *TZI* transition zone index; *TZV* transition zone volume

^a^The values obtained by subtracting the post-operative values from the pre-operative values

### Association of predictors with the difference in perioperative IPSS and retreatment in the group without prior BPH medication

In the multiple linear regression analysis, RV (*β* 0.257, 95% CI 0.092–0.421, *p* = 0.003) and RV/TZV (*β* 8.342, 95% CI 2.451–14.234, *p* = 0.006) were significantly associated with differences in perioperative IPSS (Table 4). In the multivariate logistic regression analysis to determine the correlation between the variables analyzed in Table 4 and retreatment, only RV/PV showed significant correlation (odds ratio: 64.01, 95% CI interval: 2.654–1543.794, *p* = 0.010). The preoperative total IPSS and QoL scores were also not associated with retreatment in the logistic regression analysis. RV showed a weak association with retreatment prevention; however, this was statistically insignificant (odds ratio = 1.029, 95% CI = 0.996–1.062, *p* = 0.084).

**Table 4:** Correlation between peri-operative predictors and the difference of peri-operative IPSS in the group without prior BPH medication

	Simple linear regression		Multiple linear regression		
	B (95% CI)	*p*	B (95% CI)	*p*	VIF
Age	− 0.319 (− 0.577 to − 0.060)	0.016			
PSA	0.038 (− 0.021 to 0.097)	0.208			
PV	0.038 (− 0.041 to 0.117)	0.344			
TZV	0.042 (− 0.063 to 0.148)	0.427			
Transition zone index	0.189 (− 17.123 to 17.501)	0.983			
IPP	− 0.330 (− 1.438 to 0.778)	0.554			
PUA	0.070 (− 0.060 to 0.199)	0.290			
PUL	0.014 (− 0.174 to 0.203)	0.881			
Qmax	− 0.210 (− 0.499 to 0.080)	0.154			
Voided volume	− 0.006 (-0.024 to 0.011)	0.463			
PVR	0.006 (− 0.013 to 0.026)	0.516			
Resection volume	0.184 (0.048 to 0.320)	0.008	0.257 (0.092–0.421)	0.003	1.008
Resection volume/PV	7.326 (− 1.688 to 16.340)	0.110			
Resection volume/TZV	8.668 (2.439–14.896)	0.007	8.342 (2.451–14.234)	0.006	1.028

*BPH* benign prostatic hyperplasia; *IPP* intravesical prostatic protrusion; *IPSS* International Prostate Symptom Score; *PSA* prostatic-specific antigen; *PUA* prostatic urethral angle; *PUL* prostatic urethral length; *PV* prostate volume; *PVR* post-void residual volume; *Qmax* peak flow rate; *TZV* transition zone volume; *VIF* variance inflation factor

### Cut-off values for IPSS improvement after surgery for BPH in the group without prior BPH medication

In patients whose IPSS scores decreased after surgery, the areas under the receiver operating characteristic (ROC) curve for the RV, RV/PV, and RV/TZV were 0.721, 0.667, and 0.650, respectively (*p* = 0.003*, p* = 0.024*, p* = 0.043). The evaluated cut-off values were 15.50 cm^3^, 0.33, 0.54, retrospectively. The ROC curves for retreatment were not statistically significant.

### Baseline patient characteristics in the group with prior BPH medication and association of predictors with the difference in perioperative IPSS and retreatment

The baseline patient characteristics, according to the severity of the total IPSS, are presented in Table 5. A total of 198 patients underwent retreatment within the follow-up period. There were no differences in any variables between the groups with and without retreatment. The median duration from the first surgery to retreatment was 171.0 (47.25–409.00) days. In the group without retreatment, the median follow-up period was 1011.2 (493.77–1510.34) days. When comparing the difference in preoperative IPSS and retreatment, between the groups with or without prior BPH medication, there were no statistically significant differences for each variable.

**Table 5:** Baseline characteristics of the patients according to the severity of IPSS in the group with prior BPH medication

	Total	Mild	Moderate	Severe	*p*
No. of patients	1054	202 (19.17)	571 (54.17)	281 (26.66)	
Age (years)	69.41 ± 8.95	67.81 ± 8.11	72.41 ± 9.12	71.12 ± 6.30	0.053
PSA (ng/ml)	3.34 ± 2.71	3.50 ± 1.73	3.46 ± 3.61	3.30 ± 2.12	0.523
IPSS–total	17.56 ± 7.29	4.52 ± 1.34	13.19 ± 3.24	27.13 ± 5.12	< 0.001
IPSS–QoL	3.87 ± 2.02	1.68 ± 1.49	4.07 ± 1.99	4.98 ± 1.03	< 0.001
TRUS derived parameters					
PV (cm^3^)	61.23 (41.34–82.34)	58.35 (42.12–83.20)	61.72 (41.43–78.26)	61.89 (45.23–82.37)	0.231
TZV (cm^3^)	37.39 (24.27–53.23)	37.14 (23.21–50.61)	38.11 (22.34–51.35)	37.91 (23.41–50.20)	0.879
TZI	0.61 (0.52–0.67)	0.64 (0.54–0.67)	0.62 (0.49–0.69)	0.61 (0.47–0.68)	0.786
IPP (mm)	3.82 ± 1.90	3.78 ± 2.00	3.82 ± 1.89	3.71 ± 3.11	0.659
PUA (°)	137.36 ± 14.88	140.32 ± 15.14	137.01 ± 14.67	139.20 ± 14.79	0.524
PUL (mm)	50.99 ± 11.27	52.15 ± 12.83	49.82 ± 11.10	49.15 ± 10.98	0.212
UFR derived parameters					
*Q*max (mL/sec)	10.21 ± 5.95	9.33 ± 7.33	10.53 ± 3.99	8.89 ± 6.11	0.341
Voided volume (mL)	162.23 ± 127.21	170.11 ± 122.88	158.53 ± 129.08	169.34 ± 127.00	0.864
PVR (mL)	102.83 ± 102.32	103.19 ± 101.42	102.68 ± 108.11	102.96 ± 99.23	0.784
Resection volume (cm^3^)	19.34 (11.68–24.83)	19.53 (12.00–23.42)	19.86 (11.15–24.23)	19.84 (11.58–25.50)	0.884
Resection volume/PV	0.32 (0.27–0.42)	0.33 (0.18–0.43)	0.32 (0.25–0.47)	0.32 (0.29–0.46)	0.281
Resection volume/TZV	0.51 (0.43–0.70)	0.53 (0.42–0.67)	0.52 (0.43–0.71)	0.52 (0.43–0.70)	0.696
Difference^a^					
Hb (g/dL)	0.56 ± 0.79	0.55 ± 0.97	0.54 ± 0.78	0.57 ± 1.18	0.899
Platelet (10^3^/μ/L)	22.84 ± 28.14	23.14 ± 27.76	24.19 ± 28.11	19.44 ± 17.35	0.105
Sodium (mmol/L)	− 0.43 ± 1.98	− 0.37 ± 1.45	− 0.45 ± 2.51	− 0.41 ± 3.01	0.156
Potassium (mmol/L)	0.03 ± 0.57	− 0.11 ± 1.21	0.08 ± 1.34	− 0.03 ± 0.58	0.733
IPSS–total	7.31 ± 5.24	1.81 ± 3.90	3.49 ± 7.00	11.81 ± 9.62	< 0.001
IPSS–QoL	1.35 ± 1.55	0.31 ± 1.68	1.40 ± 2.36	2.37 ± 1.32	< 0.001

Data are presented as number (%), mean ± standard deviation, and median (interquartile range)

*BPH* benign prostatic hyperplasia; *Hb* hemoglobin; *IPP* intravesical prostatic protrusion; *IPSS* International Prostate Symptom Score; *PSA* prostatic-specific antigen; *PUA* prostatic urethral angle; *PUL* prostatic urethral length; *PV* prostate volume; *PVR* post-void residual volume; *QoL* Quality of life; *Qmax* peak flow rate; *TZI* transition zone index; *TZV* transition zone volume

^a^The values obtained by subtracting the post-operative values from the pre-operative values

In the multiple linear regression analysis, RV (*β* 0.178, 95% CI − 0.032 to 0.218, *p* = 0.078) and RV/PV (*β* 7.773, 95% CI − 0.831 to 12.551, *p* = 0.091) showed weak association with differences in perioperative IPSS. In the multivariate logistic regression analysis to determine the correlation between the variables and retreatment, RV showed a marginally statistically significant association with retreatment prevention (odds ratio = 1.338, 95% CI = 0.971–0.993, *p* = 0.095).

## Discussion

Herein, we showed that RV-derived variables were related to improved perioperative IPSS and could affect future retreatment. In recent years, bipolar TURP has become widely performed, the limitation of operation time due to post-TURP syndrome has been relatively resolved compared to the past. Now, we have the conditions to perform maximal TURP with sufficient operative time. Although the results of maximal TURP in the group with prior BPH medications showed weak associations, this is likely due to the presence of very heterogeneous data. The analysis was conducted based on data from patients who were taking various medications at different doses. However, the results were clear in the group without prior BPH medication. Our results highlight the benefits of maximal TURP and may provide an opportunity for physicians to focus on it.

TURP is traditionally considered the gold standard treatment for benign prostatic obstruction and is the most frequently and widely used surgical method for the same. Under general or spinal anesthesia, operation generally requires 1–2 h, with a postoperative hospitalization period of 2–3 days [15]. Morbidity is known to occur in 5–30% of cases, and intraoperative complications include uncontrolled bleeding, dilutional hyponatremia caused by post-TURP syndrome, and acute renal failure, etc. [16]. Early postoperative complications include hematuria or infection, while late postoperative complications include incontinence (< 1%), urethral stricture (< 10%), and retrograde ejaculation (66–86%) [17, 18]. Furthermore, incidence rates of post-TURP syndrome and hemorrhage have decreased since the introduction of bipolar TURP owing to recent technological advances, which also led to availability of more surgical time and greater RV [19].

Due to technological advancements, several procedures or surgical methods have recently been developed to replace TURP. These include laser enucleation, such as HoLEP, and MISTs, such as PUL, water vapor thermal therapy, and PAE. Similar to simple prostatectomy, HoLEP involves resection of the bladder neck to the middle and lateral lobes from the verumontanum and removal of the resected tissue by mocellation in the bladder. During the mocellation procedure, care must be taken to avoid injury to the bladder walls. However, the mocellation procedure may not be easy for very large prostates. In the case of PUL, improvements in the IPSS or Qmax have been shown, but it is undeniably less effective than surgery [20]. Additionally, it has been reported that PAE has a higher retreatment rate than TURP [21]. Although water vapor thermal therapy has a lower frequency of retrograde ejaculation than TURP, it is not yet widely available and has a high cost. Furthermore, the current best evidence of the comparative effectiveness of newer MISTs is limited to a few trials with methodological flaws [22]. Therefore, the importance of TURP remains, and we believe that physicians should be rigorously trained to perform maximal TURP.

Several studies have compared the outcomes of laser enucleation and TURP. Alexander et al. compared the outcomes of HoLEP and TURP in 2022. HoLEP showed better outcomes regarding IPSS and QoL improvement than TURP. However, the operation time was longer, and it was unclear whether maximal TURP was performed; HoLEP had twice as much RVs as TURPs (41cm^3^ vs. 20 cm^3^, *p* < 0.001) and higher RV/PV (0.75 vs. 0.46, *p* < 0.001) [23]. In a network meta-analysis reported in 2022, the improvement in postoperative IPSS or Qmax of holium and thulium laser enucleation of the prostate was not statistically significant compared with that of TURP, and there were no differences in RVs in this study [24]. If the RVs in the report by Alexander et al. were similar in both groups, it was expected that there would be no difference in outcomes.

Several previous studies reported no differences in outcomes between maximal and minimal TURP [9, 10]. Herein, direct comparison was difficult because our study design was different from that of previous reports; however, in a report by Aagaard et al., the RV of maximal TURP was 15 cm^3^, which was relatively small [9]. The RV of maximal TURP in another study was relatively large at 24 cm^3^; however, the follow-up period was short (< 6 months), and the authors also mentioned that a relatively small resection ratio could induce rapid adenoma tissue regrowth [10].

Few recent studies have reported the outcomes of TURP according to RV; however, only one was reported recently, in 2017. In that study, patients were divided into three groups as per RV (Group A < 20 cm^3^, Group B 20–30cm^3^, Group C > 30 cm^3^), and preoperative IPSSs were evaluated and compared (27.0, 28.9, 28.3; Group A vs. B: *p* = 0.158, Group B vs. C: *p* = 0.546), which were not significantly different; however, postoperative IPSS showed significant differences in IPSSs between the groups B and C(9.6, 6.4, 3.3; Group A vs. B: *p* = 0.165, Group B vs. C: *p* = 0.013) [25]. This finding is consistent with our results.

We analyzed the effect of RV-derived parameters on outcomes of TURP and confirmed the importance of maximal TURP. The resulting areas under the ROC curves were analyzed and cut-off values were calculated. However, our study has several limitations. First, although retrospective data were collected for a long time by selecting a group of patients who received the first treatment, the number of patients was small. In addition, the number of physicians who performed the operations was high, and during that time, changes in the physicians’ experience and equipment could not be considered. The ultrasound equipment was also changed several times at each institution, and the physicians performing TRUS also changed several times; however, bias was not considered. Second, there were many cases in which preoperative and postoperative data were incomplete. Third, the RV was based on the weight reported by pathology, but it could not be confirmed whether the weight was measured in the same manner in both institutions. It would be interesting to compare the results of maximal TURP and HoLEP using more systematic prospective data.

## Conclusions

Despite advances in technology, such as laser enucleation and MISTs, TURP is still the most widely performed surgical technique for BPH. Recently, studies comparing maximal TURP and minimal TURP using advanced equipment have been rare, but we confirmed the importance of maximal TURP. Because maximal TURP improves postoperative outcomes and reduces retreatment rate, it may gradually become a requirement rather than an option.

## Data Availability

The datasets used and analyzed during the current study are available from the corresponding author on reasonable request.
